# Supplementary material for: A mechanism for the activation of the mechanosensitive Piezo1 channel by the small molecule Yoda1
Source: Nat Commun. 2019 Oct 3;10:4503. doi: 10.1038/s41467-019-12501-1 (PMC6776524; doi:10.1038/s41467-019-12501-1)
Supplement: Supplementary file 1 — Supplementary Information [file 41467_2019_12501_MOESM1_ESM.docx]

**A Mechanism for the Activation of the Mechanosensitive Piezo1 Channel by the Small Molecule Yoda1**

Botello-Smith et al.

**Supplementary Figures**


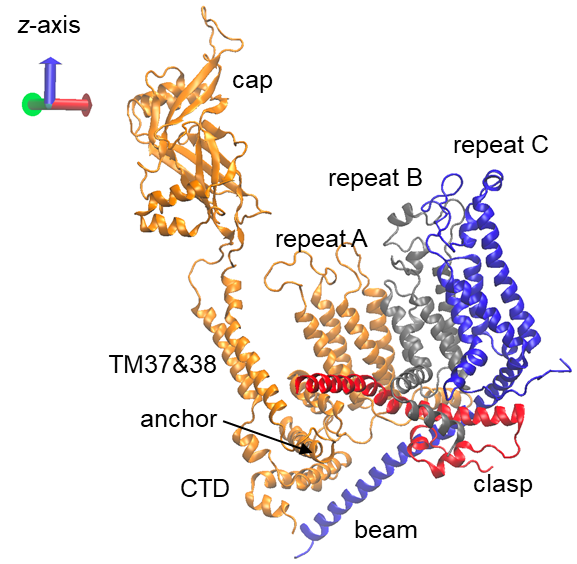


**Supplementary Figure 1:** **Computational model used in this study.** The cartoon shows our equilibrated Piezo1 model after equilibration and prior ANTON2 simulation and Yoda1 addition. For clarity, only one subunit is shown. Important structural features are indicated. TM37/38 represent the pore helices.


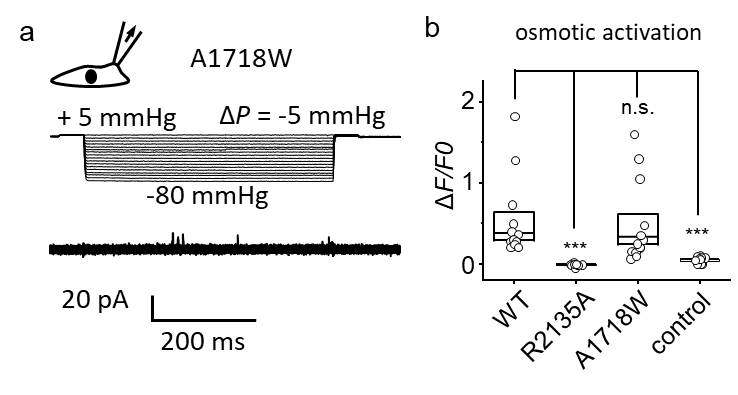


**Supplementary Figure 2: Properties of Piezo1 mutants. a,** Example of pressure-clamp cell-attached recordings for ΔPZ1 cells expressing the A1718W mutant. No pressure-elicited inward transient current was detected in fifteen tested cells. **b,** Dotted box plot showing maximal fluorescence changes obtained in ΔPZ1 cells co-expressing GC6 + WT mPZ1 (n = 14), R2135A (n = 10), A1718W (n = 13) or GC6 only (control, n = 11). The box upper and lower limits represent standard error of mean values (indicated as horizontal inner lines). Data are from n analyzed individual cells. Source data are provided as a Source Data file. Comparison of mean values between WT and mutant/control was done using two-tails Mann Whitney U tests. Asterisks indicate standard p-value range. *: 0.01 < p < 0.05; **: 0.00 1 < p < 0.01; ***: p < 0.001 and n.s. (non-significant): p > 0.05.

**
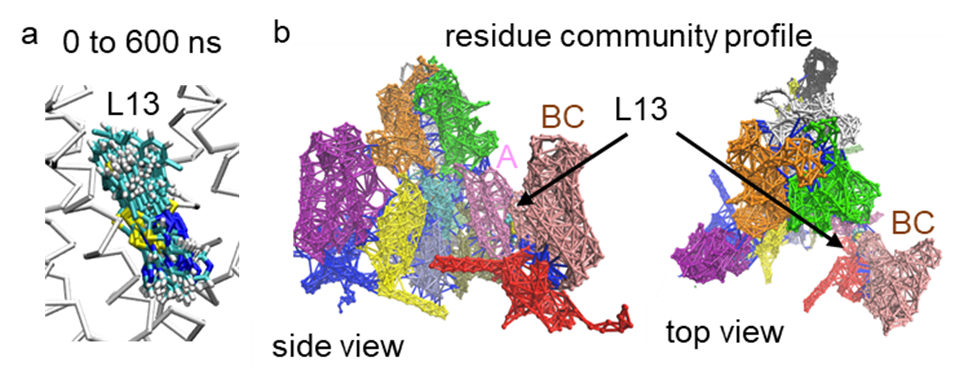
**

**Supplementary Figure 3: Yoda1 binds between two residue communities. a,** Time overlap of the bound L13 molecule along a 600 ns single-ligand trajectory. The backbone is shown at t = 600 ns. **b,** Piezo1 residue community profile calculated from a single-ligand trajectory (left: side view, right: top view). Each community is shown in different color. BC: community encompassing Piezo repeats B and C. A: Community formed by Piezo repeat A.

**Supplementary Notes**

**Supplementary Note 1**

List of primers used in this study:

| **mutation** | **method** | **forward primer** | **reverse primer** |
| --- | --- | --- | --- |
| **A1326W** | Gibson | CTCTACAATGCAtggAACCTGAAGAGC | GCTCTTCAGGTTccaTGCATTGTAGAG |
| **S1330W** | Gibson | CAGCCAACCTGAAGtggATCAACTTCCATCG | CGATGGAAGTTGATccaCTTCAGGTTGGCTG |
| **V1533W** | Gibson | CACCATGAGCGATtggCTGTGCGCAGAGC | GCTCTGCGCACAGccaATCGCTCATGGTGC |
| **A1718G** | Gibson | GTGTTCCTGTGGgggATGCTGACCATCCC | GGGATGGTCAGCATcccCCACAGGAACAC |
| **A1718I** | Gibson | GTGTTCCTGTGGatcATGCTGACCATCCC | GGGATGGTCAGCATgatCCACAGGAACAC |
| **A1718L** | Gibson | GTGTTCCTGTGGctcATGCTGACCATCCC | GGGATGGTCAGCATgagCCACAGGAACAC |
| **A1718V** | Gibson | GTGTTCCTGTGGgtcATGCTGACCATCCC | GGGATGGTCAGCATgacCCACAGGAACAC |
| **A1718W** | Gibson | GTGTTCCTGTGGtggATGCTGACCATCCC | GGGATGGTCAGCATccaCCACAGGAACAC |
| **A1972W** | Gibson | CACAAAGTACCGGtggGCCACCGACGTC | GACGTCGGTGGCccaCCGGTACTTTGTG |
| **A1973W** | Gibson | CAAAGTACCGGGCGtggACCGACGTCTACG | CGTAGACGTCGGTccaCGCCCGGTACTTTG |
| **A2091W** | Gibson | GTGCATTTACTTTtggCTGTCCGCCTA | TAGGCGGACAGccaAAAGTAAATGCAC |
| **A2094D** | Gibson | CTTTGCCCTGTCCgatTACCAGATCCGC | GCGGATCTGGTAatcGGACAGGGCAAAG |
| **A2094F** | Gibson | CTTTGCCCTGTCCttcTACCAGATCCGC | GCGGATCTGGTAgaaGGACAGGGCAAAG |
| **A2094V** | Gibson | CTTTGCCCTGTCCgttTACCAGATCCGC | GCGGATCTGGTAaacGGACAGGGCAAAG |
| **A2094W** | Gibson | CTTTGCCCTGTCCtggTACCAGATCCGC | GCGGATCTGGTAccaGGACAGGGCAAAG |
| **R2135A** | Q5 | GGTGGAGCTGgcgGCCGTCATGG | AGGAACGGCACTAGACGG |

| **backbone forward primer (anneals T7 promoter) for Gibson Assembly only** | GGAGACCCAAGCTGGCTAGC |
| --- | --- |
| **backbone reverse primer (anneals T7 promoter) for Gibson Assembly only** | GCTAGCCAGCTTGGGTCTCC |

**Supplementary Note 2**

**Procedure for automated image analysis**

The following script works best with image stacks exported from ImageJ (Save As -> Image Sequence -> Format TIFF, Start At 0, Digits 4). This will produce a series of .tif images numbered sequentially from 0000.

Run the following script in Matlab (tested in R2018a) under Editor -> Run.

You will be prompted for a .tif image. Navigate to the folder containing images of interest and select images in the set.

Enter the number of frames in the following prompt (60 for the examples given).

At this point, an error will be returned if a wrong file is selected, or if the number entered is larger than the total number of frames (e.g. entering 60 frames when the image set contains 40 files). If the frame number is smaller, only the frames up to that number will be considered.

The script will then go to the last file of the sequence and attempt to segment it using Otsu's method (as adapted from https://blog.pedro.si/2014/04/basic-cell-segmentation-in-matlab.html). This includes a "cleaning-up" step that removes regions that are smaller than 100 pixels in total. If you expect your regions to be below this threshold (e.g. because of low resolution or smaller cells), you can lower the value in "bw3 = bwareaopen(bw2, 100);".

It will then draw perimeters around the shapes it has detected (upper-left image in resulting figure) and measure the Δ*F/F_0_* values in each perimeter for each frame (upper-right image). The standard deviation of the first 20 frames will be used as the threshold for quantifying regions as active (i.e. exhibiting significant change to fluorescence intensity) or inactive. If your stimulus does not occur within the twenty-frame window, you can change the value in "twentyframes([20:framelimit],:) = [];".

The default standard is two standard deviations above or below the threshold. If you prefer a more stringent or relaxed threshold, you can increase or decrease the value in "twosigmas = 2*std(twentyframes);". You can also set a scalar threshold (e.g. twosigmas = 0.1 would consider any region that sees a 10% or higher fluorescence increase as active).

The script will then measure the percentile (lower-left) and average Δ*F/F_0_* values (lower-right) of activated regions in each frame. It will also read out the maxima in these two graphs, i.e. peak activation rate and mean intensity for activated regions.

clear all;

% Prompts for a set of .tif images corresponding to each frame of the video

% of interest. Images should be sequentially numbered (e.g. testimage01.tif

% through testimage59.tif).

[file,folder]=uigetfile('*.tif', 'Please select an initial frame');

prompt = fullfile(folder,file);

% Prompts the number of frames in the image series (0-99) and identifies

% the last frame.

lastimageid = input('Please enter the number of frames: ');

lastimageid = lastimageid - 1;

imageidstr = num2str(lastimageid);

imageidstr = ['00',imageidstr];

basename = prompt(1:end-8);

initialname = [basename,imageidstr,'.tif'];

% Converts the last frame to grayscale, passes it through contrast

% enhancement and noise removal filters.

I = imread(initialname)

I = rgb2gray(I);

I = adapthisteq(I);

I = wiener2(I, [5 5]);

% Defines an intensity threshold using Otsu's method, fills in the borders

% formed by thresholding, and removes any region below 100 pixels in size

% (should be adjusted depending on image resolution/ROI size).

bw = im2bw(I, graythresh(I));

bw2 = imfill(bw,'holes');

bw3 = bwareaopen(bw2, 100);

% Shows the border and identity of each ROI (Figure 1A).

bw3_perim = bwperim(bw3);

overlay1 = imoverlay(I, bw3_perim, [1 .3 .3]);

L = bwlabel(bw3);

s = regionprops(L, 'Centroid');

figure

plot1 = subplot (2,2,1);

imshow(overlay1)

hold on

for k = 1:numel(s)

c = s(k).Centroid;

text(c(1), c(2), sprintf('%d', k), 'HorizontalAlignment', 'center', 'VerticalAlignment', 'middle', 'color', 'g');

end

% Creates an A x B matrix, with A as the number of ROIs and B as the number

% of frames.

[columns,rows] = size(s(:,1));

framelimit = lastimageid + 1;

intensityfull=zeros(columns,framelimit);

hold off

% Fills the matrix by calculating the mean intensity of each ROI at each

% frame of the image set.

for imid = 0:lastimageid;

imstr=num2str(imid);

while(length(imstr)<4)

imstr=['0',imstr];

end;

filename = [basename,imstr,'.tif'];

Ianalysis = imread(filename);

Ianalysis = rgb2gray(Ianalysis);

intensities = regionprops(L, Ianalysis, 'MeanIntensity');

fullid = imid + 1

A = struct2cell(intensities);

out = cat(2,A{:});

intensityfull(1:columns,fullid)=out

end

% Determines deltaF/F by subtracting the baseline intensity (at t = 0) from

% each region, and dividing that result by the baseline. Plots the

% progression of deltaF/F over time (Figure 1B).

final = intensityfull';

background = bsxfun(@minus,final,final(1,:));

foverf0 = bsxfun(@rdivide,background,final(1,:));

plot2 = subplot (2,2,2);

plot(foverf0)

set(gca,'XLim',[0 lastimageid])

ylabel('Fold change in intensity');

xlabel('Frame number');

title('\rmIntensity fluctuations');

% Determines an activation threshold by averaging the deltaF/F values for

% the first twenty frames (adjustable - twenty is used here to encompass

% 10s before and after Yoda1 addition).

twentyframes = foverf0;

twentyframes([20:framelimit],:) = [];

% Sets each frame as active if they're at least two standard deviations

% above the baseline, and inactive otherwise.

twosigmas = 2*std(twentyframes);

binaryactivation = foverf0

binaryactivation(binaryactivation<binaryactivation(1,:)+twosigmas)=0

binaryactivation(binaryactivation>binaryactivation(1,:)+twosigmas)=1

binaryactivation(binaryactivation==binaryactivation(1,:)+twosigmas)=1

% Determines the mean deltaF/F for activated ROIs at each frame (Figure 1C).

activationmask = binaryactivation.*foverf0;

activationmean = sum(activationmask,2) ./ sum(activationmask~=0,2)

activationmean(isnan(activationmean))=0;

activationmean = activationmean;

plot3 = subplot (2,2,3);

plot(activationmean);

set(gca,'XLim',[0 lastimageid])

limity = max(activationmean) + 0.05;

ylim([-0.1 limity])

ylabel('Fold change in average intensity of activated cells');

xlabel('Frame number');

title('\rmIntensity profile');

% Determines the percentile of activated ROIs at each frame (Figure 1D).

binariessum = sum(binaryactivation,2);

binariessum = 100.*binariessum./columns;

plot4 = subplot (2,2,4);

plot(binariessum);

ylim([-5 105]);

set(gca,'XLim',[0 lastimageid])

ylabel('Percentile of activated ROIs');

xlabel('Frame number');

title('\rmNumber of activations');

set(0, 'Units', 'normalized');

set(plot2, 'Units', 'normalized');

set(plot3, 'Units', 'normalized');

set(plot1, 'Position', [0.09, .54, .4, .45]);

set(plot2, 'Position', [0.52, .60, .4, .35]);

set(plot3, 'Position', [0.12, .12, .35, .35]);

set(plot4, 'Position', [0.52, .12, .4, .35]);

% Prints the maxima in percentile activation and average deltaF/F over the

% entire frame range.

maxactivation = max(binariessum);

maxintensity = max(activationmean);

output1 = sprintf('The maximum percentage of activated cells is %g%%.',maxactivation)

output2 = sprintf('The average intensity peaks at %g-fold compared to baseline.',maxintensity)
