# Supplementary material for: A mechanism for the activation of the mechanosensitive Piezo1 channel by the small molecule Yoda1
Source: Nat Commun. 2019 Oct 3;10:4503. doi: 10.1038/s41467-019-12501-1 (PMC6776524; doi:10.1038/s41467-019-12501-1)
Supplement: Supplementary file 7 — Reporting Summary [file 41467_2019_12501_MOESM7_ESM.pdf]

nature research

Corresponding author(s): NCOMMS-19-04201A

Last updated by author(s): Aug 8, 2019

Reporting Summary

Nature Research wishes to improve the reproducibility of the work that we publish. This form provides structure for consistency and transparency in reporting. For further information on Nature Research policies, see Authors & Referees and the Editorial Policy Checklist.

Statistics

For all statistical analyses, confirm that the following items are present in the figure legend, table legend, main text, or Methods section.

n/a	Confirmed	
☐	☒	The exact sample size (*n*) for each experimental group/condition, given as a discrete number and unit of measurement
☐	☒	A statement on whether measurements were taken from distinct samples or whether the same sample was measured repeatedly
☐	☒	The statistical test(s) used AND whether they are one- or two-sided<br>*Only common tests should be described solely by name; describe more complex techniques in the Methods section.*
☒	☐	A description of all covariates tested
☒	☐	A description of any assumptions or corrections, such as tests of normality and adjustment for multiple comparisons
☐	☒	A full description of the statistical parameters including central tendency (e.g. means) or other basic estimates (e.g. regression coefficient) AND variation (e.g. standard deviation) or associated estimates of uncertainty (e.g. confidence intervals)
☐	☒	For null hypothesis testing, the test statistic (e.g. *F*, *t*, *r*) with confidence intervals, effect sizes, degrees of freedom and *P* value noted<br>*Give P values as exact values whenever suitable.*
☒	☐	For Bayesian analysis, information on the choice of priors and Markov chain Monte Carlo settings
☒	☐	For hierarchical and complex designs, identification of the appropriate level for tests and full reporting of outcomes
☒	☐	Estimates of effect sizes (e.g. Cohen's *d*, Pearson's *r*), indicating how they were calculated

Our web collection on statistics for biologists contains articles on many of the points above.

Software and code

Policy information about availability of computer code

Data collection	pemed.CUDA module of AMBER16<br>MD simulation engine on ANTON2 supercomputer<br>Nikon NIS Element
Data analysis	cpptraj program from the AMBER suite<br>VMD1.9.3<br>GraphPad Prism 7<br>Origin Pro 2018<br>MATLAB (script available as Supplementary Note 2)

For manuscripts utilizing custom algorithms or software that are central to the research but not yet described in published literature, software must be made available to editors/reviewers. We strongly encourage code deposition in a community repository (e.g. GitHub). See the Nature Research guidelines for submitting code & software for further information.

Data

Policy information about availability of data

All manuscripts must include a data availability statement. This statement should provide the following information, where applicable:

- Accession codes, unique identifiers, or web links for publicly available datasets
- A list of figures that have associated raw data
- A description of any restrictions on data availability

Experimental and computational data are available upon reasonable request. Requests should be made to Jerome Lacroix (jlacroix@westernu.edu) or to Yun Luo (luoy@westernu.edu).

Field-specific reporting

Please select the one below that is the best fit for your research. If you are not sure, read the appropriate sections before making your selection.

☒ Life sciences ☐ Behavioural & social sciences ☐ Ecological, evolutionary & environmental sciences

For a reference copy of the document with all sections, see nature.com/documents/nr-reporting-summary-flat.pdf

Life sciences study design

All studies must disclose on these points even when the disclosure is negative.

Sample size	No sample size calculation was performed.
Data exclusions	No experimental data were excluded from analyzes.
Replication	Most attempts to replicate experimental results were successful. No replication was attempted for our simulation data.
Randomization	No randomization of sample was performed.
Blinding	Experimental and Simulation Data were acquired using softwares, not by human intervention, thus no blinding was necessary for data collection.

Reporting for specific materials, systems and methods

We require information from authors about some types of materials, experimental systems and methods used in many studies. Here, indicate whether each material, system or method listed is relevant to your study. If you are not sure if a list item applies to your research, read the appropriate section before selecting a response.

Materials & experimental systems

n/a	Involved in the study	
☒	☐	Antibodies
☐	☒	Eukaryotic cell lines
☒	☐	Palaeontology
☒	☐	Animals and other organisms
☒	☐	Human research participants
☒	☐	Clinical data

Methods

n/a	Involved in the study	
☒	☐	ChIP-seq
☒	☐	Flow cytometry
☒	☐	MRI-based neuroimaging

Eukaryotic cell lines

Policy information about cell lines

Cell line source(s)	HEK293T cells lacking Piezo1 (Delta PZ1) were created by and obtained from the Patapoutian Group at Scripps Research.
Authentication	None of the cell lines were authenticated.
Mycoplasma contamination	None of the cell lines were tested for Mycoplasma contamination.
Commonly misidentified lines (See ICLAC register)	None of the cells are known to be commonly misidentified.
