# Supplementary material for: A mechanism for the activation of the mechanosensitive Piezo1 channel by the small molecule Yoda1
Source: Nat Commun. 2019 Oct 3;10:4503. doi: 10.1038/s41467-019-12501-1 (PMC6776524; doi:10.1038/s41467-019-12501-1)
Supplement: Supplementary file 8 — Description of Additional Supplementary Files [file 41467_2019_12501_MOESM8_ESM.pdf]

Title: Supplementary Movie 1: Sampling of Piezo1 by Yoda1 ligands.
Description: Multi-ligand trajectory (top view, 0 to 4.8 µs, sampled every 24 ns) showing protein backbone in white surface, phosphate groups as dots and each Yoda1 ligand (Van der Waals representation) in a unique color.

Title: Supplementary Movie 2: Activation of the R2135A mutant with Yoda1.
Description: ΔPZ1 cells co-expressing GCaMP6m and the R2135A mutant were imaged for one minute at 1 frame/sec under epifluorescence illumination. 100µM Yoda1 was added at t = 10s. The images are displayed at 1 frame s^{-1}.

Title: Supplementary Movie 3: Stretch-induced Piezo1 motions, side view.
Description: Multi-ligand trajectory (side view, 0 to 6.9 µs, sampled every 24 ns) showing protein backbone (new cartoon, silver), phosphate groups (yellow spheres) and the L13 ligand (blue, Van der Waals representation).

Title: Supplementary Movie 4: Stretch-induced Piezo1 motions, top view.
Description: Multi-ligand trajectory (top view, 0 to 6.9 µs, sampled every 24 ns) showing protein backbone (new cartoon, silver), phosphate groups (yellow spheres) and the L13 ligand (blue, Van der Waals representation).

Title: Supplementary Movie 5: Tilt motion of Piezo1 arms.
Description: Projection of the top 3 principal component modes clustered using the HDBSCAN method. The centers of each cluster were then computed and exported in pdb format for visualization.
